# Respiration and Heart Rate Modulation Due to Competing Cognitive Tasks While Driving

**DOI:** 10.3389/fnhum.2018.00525

**Published:** 2019-01-07

**Authors:** Antonio R. Hidalgo-Muñoz, Adolphe J. Béquet, Mathis Astier-Juvenon, Guillaume Pépin, Alexandra Fort, Christophe Jallais, Hélène Tattegrain, Catherine Gabaude

**Affiliations:** Univ Lyon, IFSTTAR, TS2, LESCOT, Lyon, France

**Keywords:** breathing rate, cognitive workload, driving, heart rate variability, respiration

## Abstract

Research works on operator monitoring underline the benefit of taking into consideration several signal modalities to improve accuracy for an objective mental state diagnosis. Heart rate (HR) is one of the most utilized systemic measures to assess cognitive workload (CW), whereas, respiration parameters are hardly utilized. This study aims at verifying the contribution of analyzing respiratory signals to extract features to evaluate driver’s activity and CW variations in driving. Eighteen subjects participated in the study. The participants carried out two different cognitive tasks requiring different CW demands, a single task as well as a competing cognitive task realized while driving in a simulator. Our results confirm that both HR and breathing rate (BR) increase in driving and are sensitive to CW. However, HR and BR are differently modulated by the CW variations in driving. Specifically, HR is affected by both driving activity and CW, whereas, BR is suitable to evidence a variation of CW only when driving is not required. On the other hand, spectral features characterizing respiratory signal could be also used similarly to HR variability indices to detect high CW episodes. These results hint the use of respiration as an alternative to HR to monitor the driver mental state in autonomic vehicles in order to predict the available cognitive resources if the user has to take over the vehicle.

## Introduction

It has been reported that 25–50% of road accidents are due to lack of driver attention (Mosedale et al., 2004). Distraction related to external factors and inattention related to driver internal state are two phenomena that, despite their different impacts on driving activity, are responsible for an equivalent number of accidents (Galéra et al., 2012). Hence, sharing attentional resources between driving and a competing cognitive task should be considered as a major risk factor for transport safety (Harbluk et al., 2007).

According to Wickens (2002) the available attentional resources to complete a task are reduced when other perceptual tasks are concurrent. For instance, while driving, orienting attention to visual or auditory stimuli unrelated to the driving task can lead to poorer integration of environmental information and thus prevent the operator from performing the task safely (Galéra et al., 2012). Besides, the task-unrelated thoughts can also hinder driving performance reducing the lane variability and inducing intermittent steering behavior (Lemercier et al., 2014). Furthermore, different emotions such as anger can provoke mind wandering hampering the attentional focus and modulating driver’s performance (Techer et al., 2017). Therefore, the incorporation of driver’s internal state monitoring is desirable not only for manual driving evaluation. It will be needed into autonomous vehicles to improve safety during takeover phases (Eriksson and Stanton, 2017), since drivers will be able to carry out supplementary cognitive tasks when manual driving is not required.

In order to determine objective and cost-effective indicators to monitor cognitive workload (CW) variations, several physiological measures have been proposed in the literature, including brain signals (Mandrick et al., 2016). However, cardiovascular signals have been widely privileged in neuroergonomics approaches due to their simple implementation in ecological settings and sensitivity to CW levels (Ryu and Myung, 2005; Stuiver et al., 2014; Mansikka et al., 2016). Specifically for driver monitoring applications, Heine et al. (2017) measured the cardiac activity during a driving session simultaneously to the accomplishment of a secondary cognitive task. In their study, the authors showed an increase of heart rate (HR) and a decrease of HR variability (HRV) under high CW demands, modulated according to three difficulty levels. Similarly, Mehler et al. (2011) have also highlighted the robustness of HR in the comparison between a main driving task and an additional cognitive task. The authors demonstrated the possibility to distinguish a single task driving and different levels of additional CW using HR and HRV analysis. Either way, the unspecific nature of cardiac activity variations, which can be influenced by emotions among other factors like the age or the sex (Overbeek et al., 2012), makes difficult to claim properly the link between HR, HRV, and CW. The analysis of complementary measures is then highly recommended.

Though usually neglected, respiration signals are emerging as an interesting alternative to traditional cardiovascular measurements to evaluate CW (Cretikos et al., 2008; Grassmann et al., 2016). As well known, heart and respiratory rates are linked via the sinus arrhythmia, but their mutual influence differs according to breathing rate (BR) (Song and Lehrer, 2003; Laborde et al., 2017), so both measures might complementary in the drivers’ monitoring. In addition, respiration rhythms present individual pattern (Benchetrit, 2000), suggesting an advantage to implement customized interfaces to improve this monitoring.

The purpose of this study aims at exploring the advantage to incorporate BR related features, with regard to ECG features, so as to evaluate driver’s activity and CW variations. According to the conclusions extracted from the literature, an increase of HR is expected when CW level increases due to the activation of the orthosympathic system, which is also responsible of increasing arterial pressure and pupil diameter in high demanding cognitive tasks.

Inversely, a decrease of HRV parameters would be expected when the cognitive effort increases in order to ease stress regulation, except for the spectral parameter representing the sympatho-vagal balance (Lee et al., 2007). An increase of this index is expected according to a previous reliability study from Mukherjee et al. (2011), although there is no consensus on its computation and meaning to study stress and CW (Miu et al., 2009; Billman, 2011; Chanel et al., 2011).

On the other hand, given that driving consists in as a physical and cognitive task simultaneously, the impact of movements on the HR could hide the effect of the CW of an additional task, as remarked by Taelman et al. (2011), even with low demanding physical movements.

Concerning respiration, a similar pattern to HR is expected for BR. However, to our knowledge, there is not previous works dealing with the different sensitivity of HR and BR facing CW variations while driving.

## Materials and Methods

### Participants

Eighteen healthy volunteers (10 males, 22.7 ± 1.4 years) were recruited via an online procedure and participated in the study. All participants had normal auditory acuity and normal or corrected to normal vision. None of them declared any cardiorespiratory or neurological diseases. All participants were asked to sign a written consent and agreed with the absence of financial compensation. The study complied with the Declaration of Helsinki for human experimentation. A valid driving license for at least 3 years was required.

### Experimental Setting

#### Cognitive Workload Manipulation

**FIGURE 1:**
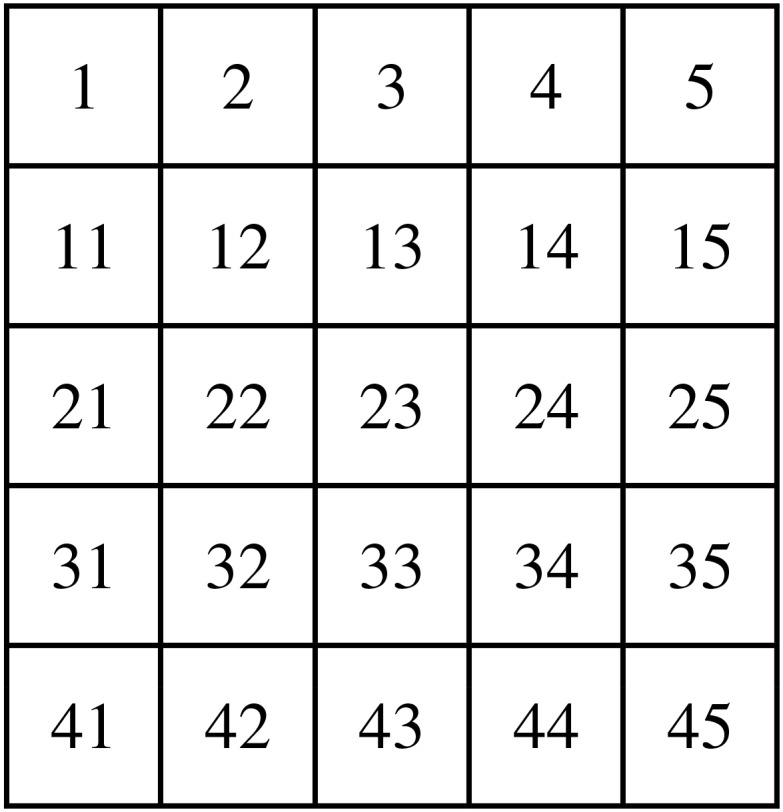
Grid memorized by the participants for the High Cognitive Workload condition.

The participants performed two different cognitive tasks, 5-min length approximately, requiring different CW demands:

- Low Cognitive Workload (LCW) condition consisting in a beep counting. Twelve beeps were randomly presented during this condition.
- High Cognitive Workload (HCW) condition consisting in a mental displacement within a previously memorized 5 × 5 numerical grid (Figure 1) from the central box (numbered 23 in the figure), according to direction instructions (up, down, left, and right) that were presented orally, and an arithmetic task consisting in adding the numerical value from the actual box to the result previously obtained. One after another, between seven and ten numbers had to be summed.

The presentation order of LCW and HCW conditions were counterbalanced among the participants.

#### Driving Scenario and Simulation

**FIGURE 2:**
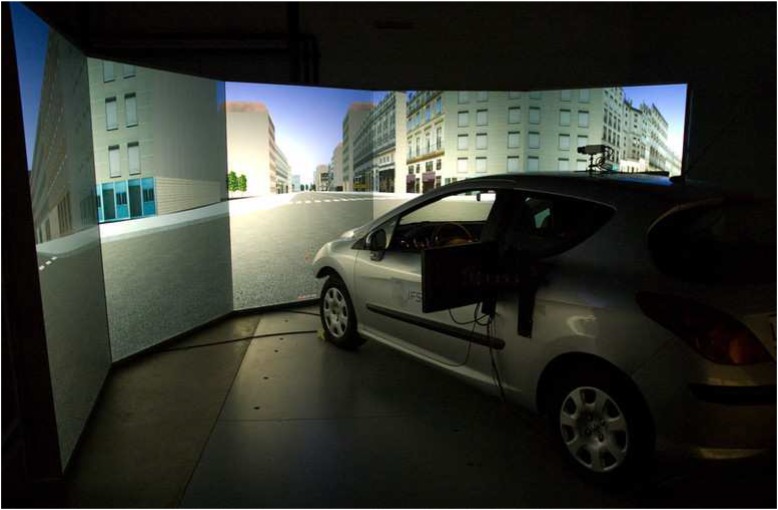
Driving simulator at IFSTTAR-LEPSIS.

The participants carried out both cognitive tasks during two activities: a Single Task (ST) being seated into the vehicle without driving as well as a Dual Task (DT) where driving is simultaneously performed, in the IFSTTAR-LEPSIS simulator (Figure 2).

The driving simulator consisted in a Peugeot 308 cabin surrounded by seven video projection screens providing a 270° horizontal and 47° vertical forward field of view. The vehicle was equipped with a manual transmission, and the steering wheel had a force feedback system. Participants drove in straight lines and curves in a peri-urban area with sparse traffic keeping a speed of 70 km/h during 4.5 min. A training block of 5 min was carried out before the DT conditions.

#### Cardiorespiratory Activity Acquisition

ECG signal was recorded (sampling rate = 1 kHz, hardware band-pass filtering 1–35 Hz) throughout the entire experiment by placing three electrodes on the clavicle, under the last left rib and on top of the right hip of the participant. The respiration signal (sampling rate = 1 kHz, hardware low-pass filtering 0–1 Hz) was recorded by using a respiratory belt transducer. Both signals were registered via a wireless physiology solution (BioNomadix system with MP150 Biopac system).

### Signal Processing

#### Electrocardiogram

The ECG signal pre-processing included an additional band-pass filtering with 0.5 and 40 Hz cut-off frequencies by a 4th-order zero-phase Butterworth filter to reject residual high frequency noise and baseline wander artifacts. Every ECG parameter was computed by HRV Analysis (Pichot et al., 2016). A visual inspection was carried out to check signal quality. Due to no exploitable signals or to acquisition issues in at least one of the conditions, the statistical analysis on ECG were conducted on 12 participants.

Besides the dominant HR, several ECG features were computed to evaluate HRV (Billman, 2011) within the 4.5 min-length condition. In order to obtain reliable data and comparable results to the ones showed in the literature, the recommendations from Laborde et al. (2017) to analyze HRV were taken into account.

- SDNN: Standard deviation of R-R interval lengths for each 5-min segment.
- RMSSD: Root mean squared of the successive differences between adjacent R-R intervals. Usually, it is associated with fast (parasympathetic) variability and it is less affected by respiratory influences (Hill et al., 2009).
- pNN20: Proportion of R-R intervals which differ more than 20 ms with respect to the adjacent previous R-R interval.
- pNN50: Proportion of R-R intervals which differ more than 50 ms with respect to the adjacent previous R-R interval.
- LF (Low Frequency): Spectral power in 0.05–0.15 Hz band, related to parasympathetic and sympathetic activity.
- HF (High Frequency): Spectral power in 0.15–0.4 Hz band, related to parasympathetic (vagal) activity.
- LF/HF: Ratio of energy in the LF and HF bands. A decrease in this score might point to either decrease in sympathetic or increase in parasympathetic tone (Hill et al., 2009).

#### Respiratory Signal

Besides the BR (inspirations/minute) from the respiratory signal, the following features were computed (Veltman and Gaillard, 1996):

- Mid-band: Spectral power in 0.07–0.14 Hz band.
- High-band: Spectral power in 0.15–0.50 Hz band.

One participant was eliminated due to technical problems for acquisition. Two additional participants were discarded due to low quality signals in terms of noise or inaccurate belt adjustment. Another participant was considered as an outlier [with values out of (mean ± 2 × SD)] for the spectral analysis. Therefore, 15 and participants were included in the respiratory signal analysis for temporal and spectral analyses, respectively.

#### Driving Performance

In order to determine whether the different levels of CW impact driving performance, several representative parameters were recorded from the vehicle. According to the task instructions, speed-related parameters were relevant to verify if the participant kept the speed as close as possible to the 70 km/h. The lateral deviation from the reference line of the trajectory has been also computed, since it is a common parameter to characterize driver behavior.

- Speed mean value (km/h)
- Speed variability (km/h)
- Lateral deviation variability (m)

In order to minimize the influence of the driving scenario position and driving style, the considered mean values take into consideration only 6 s [following the parameters employed in Pépin et al. (2017)] after every stimulus presentation and averaged for all the task duration.

### Statistical Analysis

Normality was first checked for the parameter value distributions by means of the Shapiro-Wilk’s test. Then, a two-way analysis of variance (ANOVA 2 × 2), the first factor representing the activity (ST and DT) and the second one referring to the CW demand (LCW and HCW) was performed for the statistical contrasts for every feature. HSD Tukey correction was applied for *post hoc* analysis. All the statistical analyses were carried out by using SPSS 21.0 software.

## Results

Tables 1, 2 compiles the mean value and standard deviation of every parameter included in the analysis.

**Table 1:** Cardio-respiratory parameters (mean ± SD).

	Single task		Dual task	
	LCW	HCW	LCW	HCW
HR (bpm)	77.62 ± 11.48	82.83 ± 12.24	84.29 ± 13.76	88.14 ± 15.07
SDNN (ms)	63.77 ± 25.07	62.46 ± 23.98	60.20 ± 20.17	58.19 ± 21.40
RMSSD (ms)	44.91 ± 22.42	40.29 ± 19.53	37.77 ± 18.33	33.51 ± 16.93
pNN20 (%)	56.59 ± 22.01	51.61 ± 20.32	47.32 ± 23.44	44.06 ± 23.63
pNN50 (%)	23.51 ± 18.34	19.31 ± 16.85	16.63 ± 15.96	14.35 ± 13.63
LF	1696 ± 1453	1466 ± 1445	1146 ± 985.5	1100 ± 973.2
HF	941.4 ± 844.4	739.2 ± 668.6	529.8 ± 502.2	532.6 ± 536
LF/HF	2.56 ± 1.67	2.61 ± 1.83	3.28 ± 2.12	3.41 ± 2.49
BR (BBI/min)	16.0 ± 2.7	17.2 ± 2.0	19.6 ± 2.4	19.3 ± 2.4
Mid-band^1^	189.1 ± 108.7	151.7 ± 71.5	203.8 ± 121.4	169.2 ± 105
High-band^1^	713.1 ± 120.4	736 ± 95.7	728.3 ± 127.9	759.9 ± 117.9


*BBI, breath to breath intervals; LCW, low cognitive workload; HCW, high cognitive workload. ^*1*^Computed from respiratory signals.*

**Table 2:** Driving performance indices (mean ± SD).

	LCW	HCW
Speed mean value (km/h)	78.13 ± 6.67	78.37 ± 7.31
Speed variability (km/h)	1.481 ± 0.70	1.304 ± 23.98
Lateral deviation variability (m)	0.135 ± 0.034	0.107 ± 0.025


### Cardiovascular Activity

**FIGURE 3:**
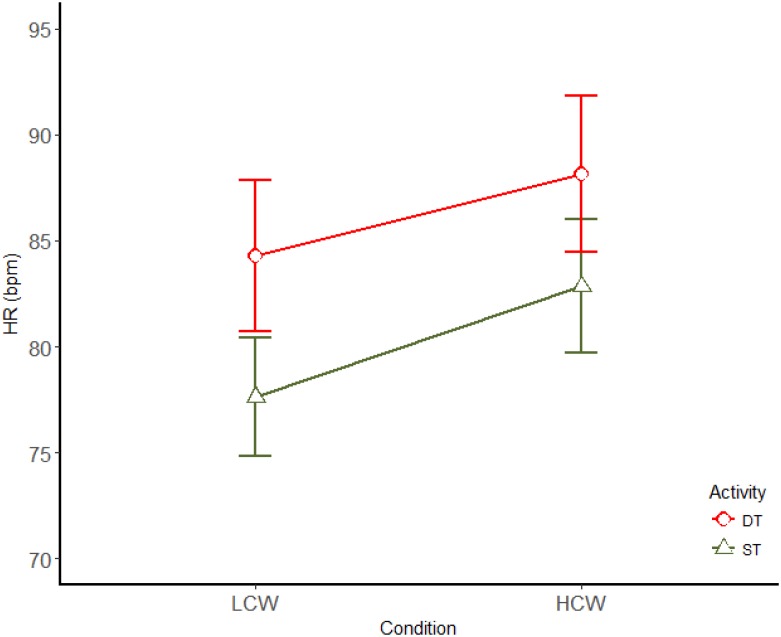
Main effects (mean ± SE) of driving and CW for HR. LCW, low cognitive workload; HCW, high cognitive workload; ST, single task; DT, dual task.

Concerning HR, a main effect of the activity [*F*(1,11) = 21.2; *p* < 0.01; η^2^ = 0.658] and CW demand between LCW and HCW condition [*F*(1,11) = 32.68; *p* < 0.01; η^2^ = 0.748] were found, showing an increase in HR while driving in comparison to ST and for HCW (Figure 3). Their interaction was not significant.

**FIGURE 4:**
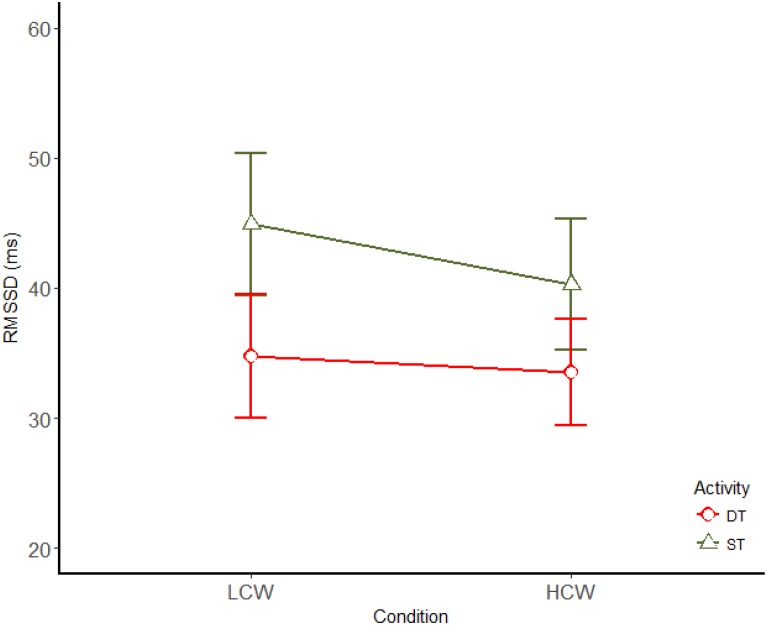
Main effects (mean ± SE) of driving and CW for RMSSD. LCW, low cognitive workload; HCW, high cognitive workload; ST, single task; DT, dual task.

Similarly, but in an opposed direction, several HRV parameters have shown significant results. RMSSD presented a main effect of the activity [*F*(1,11) = 8.6; *p* = 0.014; η^2^ = 0.439] and of the CW demand [*F*(1,11) = 7.99; *p* = 0.016; η^2^ = 0.421], showing that RMSSD decreases in driving and under HCW condition (Figure 4). pNN20 presented a main effect of the activity [*F*(1,11) = 7.7; *p* = 0.018; η^2^ = 0.413] and of the CW demand [*F*(1,11) = 13; *p* = 0.004; η^2^ = 0.543] showing that pNN20 decreases in driving and under HCW condition too. The same tendency was evident for pNN50, presenting a main effect of the activity [*F*(1,11) = 6.8; *p* = 0.024; η^2^ = 384] and of the CW demand [*F*(1,11) = 6.9; *p* = 0.023; η^2^ = 0.387]. There was no significant result for SDNN.

Concerning spectral features, LF power presented a main effect of CW demand [*F*(1,11) = 6.6; *p* = 0.026; η^2^ = 0.375] showing that LF power decreases under HCW condition. On the other side, HF power presented a main effect of the activity [*F*(1,11) = 9.4; *p* = 0.011; η^2^ = 0.461] showing that HF power decreases in driving. LF/HF ratio presented a main effect of activity [*F*(1,11) = 13.5; *p* = 0.004; η^2^ = 0.551] showing that LF/HF ratio increases in driving.

### Respiration

**FIGURE 5:**
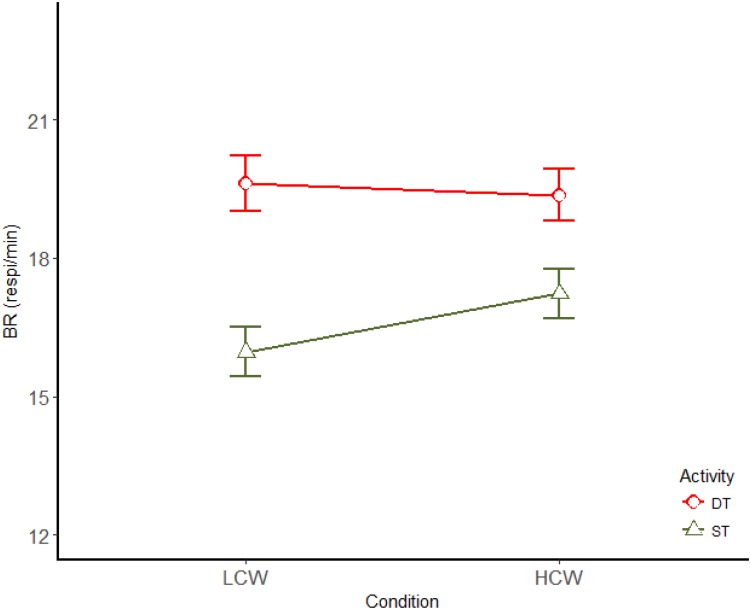
Interaction (mean ± SE) between driving and CW for BR. LCW, low cognitive workload; HCW, high cognitive workload; ST, single task; DT, dual task.

Regarding BR, this parameter increased while driving [*F*(1,14) = 48.19; *p* < 0.01; η^2^ = 0.775] and no effect of CW demand was found. Conversely to HR, an interaction between the activity and the CW demand was found for BR [*F*(1,14) = 11.55; *p* < 0.01; η^2^ = 0.452] (Figure 5). Whereas, the *post hoc* analysis showed that BR increased for HCW in comparison to LCW under ST condition (*p* < 0.01), no significant differences between LCW and HCW were found while driving.

**FIGURE 6:**
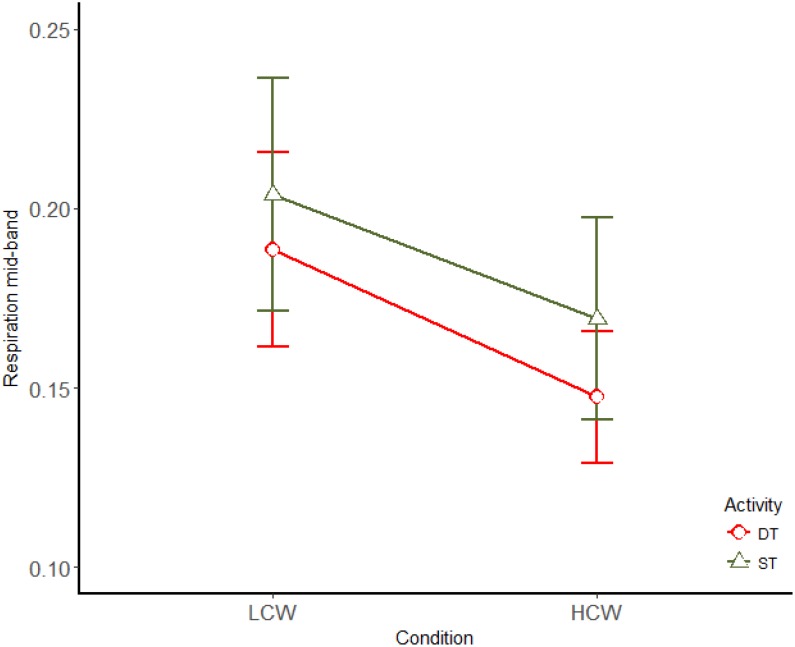
Main effects (mean ± SE) of CW for respiration mid-band. LCW, low cognitive workload; HCW, high cognitive workload; ST, single task; DT, dual task.

Concerning the spectral analysis, only a main effect of the CW demand was significant for the mid-band [*F*(1,13) = 5.947; *p* = 0.03; η^2^ = 0.314], this parameter decreased when CW increases (Figure 6). No significant differences were found for the respiration high-band.

### Driving Performance

Driving performance were analyzed in LCW and HCW condition during DT. Since only two conditions were compared, a paired Student *t*-test for each comparison was performed. Only the lateral deviation showed significant differences [*t*(16) = 3.013; *p* = 0.008] between LCW and HCW, being higher for LCW. Note that, although this parameter can be utilized to characterize driving performance, the values fell into an acceptable rang and did not permit to distinguish accurately different driving behaviors.

## Discussion

### Cardiac Activity Linked to Competing CW Variation in Driving

The HR increase in driving may be explained, at least partly, by the greater CW generated by a double-task situation (Mehler et al., 2009; Heine et al., 2017) due to the competition of working memory components between the cognitive tasks and the added driving task (Farmer et al., 1986; Wickens, 2002; Johannsdottir and Herdman, 2010). Indeed, driving is an exercise that solicits notably the visuospatial sketchpad (Johannsdottir and Herdman, 2010) which is also solicited to accomplish the additional cognitive task by mentally moving into the grid (Figure 1) for HCW condition, hampering driving performance in terms of lateral deviation variability, since the decrease of this parameter has been linked to inattention and a loss of performance (Lemercier et al., 2014). Either way, it is fair to remark that the motor activity involved in driving may also influence HR activity. However, it is not possible to isolate the modulation due to the physical activity linked to driving activity from the driving task. To this aim, a deep analysis of posture should to be carried out (see Zhao et al. (2018) for a modern method to extract posture information that could be included as co-variable in the statistical analysis).

Complementary to HR, a decrease of HRV correlates with higher CW, as shown in other research works dealing with flying simulation where the task difficulty was manipulated to vary CW demand (Durantin et al., 2014). In addition, the decrease of HRV parameters could be linked to the emotional regulation (Appelhans and Luecken, 2006) and subsequently in relation to the stress generated by the task difficulty as other research works suggest (Mandrick et al., 2016; Hidalgo-Muñoz et al., 2018). It is important to remark the importance of the counterbalancing for the different tasks, as HRV parameters are sensitive to the physical activity previous to the task realization (Luft et al., 2009).

Regarding the spectral analysis, in our experiment, the increase of LF/HF ratio is in accordance with the results obtained with pilots during assisted and unassisted flight, where arousal is modulated by the flight task complexity (Skibniewski et al., 2015). According to our results, the LF/HF increase is due to the HF decrease (diminution of the parasympathetic influence). This result is also in line with other research suggesting cardiac activity is controlled by the sympathetic system under HCW (Lean and Shan, 2012). Furthermore, Ruscio et al. (2017) have reported the suitability of this feature to predict reaction time in takeover after expected or unexpected warning signals in driving. These authors suggested that during an autonomous phase, an on-request take-over could generate an overload due to a possible shrink of attentional capacities. This would mainly involve the parasympathetic system, whereas, the sympathetic system is dominant when drivers expect the signal. Therefore, the parameters obtained from the autonomous nervous system signals can be useful to determine if the driver will be able to perform the takeover effectively.

### Respiratory Activity Linked to Competing CW Variation in Driving

Regarding the respiratory parameters, as expected, BR was the most sensitive respiratory parameter, as claiming by other works (Veltman and Gaillard, 1998). Our results show a significant increase in BR when performing a driving task. In addition, BR can also discriminate between two levels of task difficulty without driving (ST condition). Actually, BR is accelerated when CW increases, as reported in other research works (Grassmann et al., 2016). Moreover, BR was suggested to be one of the best physiological variables to classify CW when only a single variable is considered as input (Hogervorst et al., 2014). The authors modulated the CW by using N-back task. However, in their study a motor activity, such as the one involved in driving, was not considered. Of note, in our protocol, an apparent ceiling effect has been reached on the BR in simulated driving, which no longer makes it possible to discriminate different levels of CW.

Complementary respiratory parameters describing the BR variation seemed to be also useful in order to distinguish different levels of CW. Indeed, the mid-band energy of respiration could be used as indicator of CW variations, as an alternative to HRV parameters so as BR is less affected by driving task, since no main effect was evidenced. Nonetheless, contrarily to our expectations, no significant results were found for the high-band. Arguably, this parameter would be impacted by the intrinsic driving task difficulty, analogously to flight difficulty in pilots (Veltman and Gaillard, 1996). The spectral structure variations could be explained, at least partly, by the physiological resetting after responding to cognitive stimuli, as suggested by Vlemincx et al. (2010). Driving and HCW situations could elicit increasing stress and temporal pressure making breathing progressively random and its spectrum. In any case, both spectral parameters (high- and mid-band) from respiratory signals are more robust to driving activity than BR.

The incorporation of other respiratory features such as the tidal volume or other parameters linked to the signal amplitude values and entropy would be suitable to consider (Vlemincx et al., 2011) as well as end-tidal carbon dioxide concentration (Karavidas et al., 2010). Nevertheless, a different kind of sensor, including a capnograph, would be needed to assure stable measurements. On the other hand, the implementation of algorithms to extract BR from ECG recordings to evaluate CW is another possibility to explore (Moody et al., 1986; Thayer et al., 1996; Madhav et al., 2011).

## Conclusion

The present study shows that cardiac and respiratory parameters may be employed as complementary markers reflecting the mental state under different conditions. Whereas, HR and HRV are impacted by competing CW variations in single and driving dual-tasks, BR could be suitable to evidence a variation of CW when driving is not required as an alternative to HR to monitor the driver mental state in autonomous vehicles, where a continuous driving is not required, in order to predict the available cognitive resources if a take-over of the vehicle is needed or to keep the driver in the loop.

In future studies, it would be desirable to manipulate the CW level of the driving task in order to determine the influence of the complexity of driving task and the particularity of supplementary CW to driving situation to identify cognitive overload situations. The analysis of BR under different emotional conditions and moods will be also interesting in order to determine the influence of emotion on respiratory behavior in driving.

## Author Contributions

All authors contributed to writing and proof correction. GP acquired the data. AH-M, AB, and MA-J processed the signals. AB and MA-J analyzed the data. AF, CG, CJ, and GP have designed the experiments. HT, CG, and AH-M contributed to coordination.

## Conflict of Interest Statement

The authors declare that the research was conducted in the absence of any commercial or financial relationships that could be construed as a potential conflict of interest.
